# Hydrolysis with *Cucurbita ficifolia* serine protease reduces antigenic response to bovine whey protein concentrate and αs-casein

**DOI:** 10.1007/s00726-015-2013-2

**Published:** 2015-06-03

**Authors:** Konrad Babij, Joanna Bajzert, Anna Dąbrowska, Marek Szołtysik, Aleksandra Zambrowicz, Gert Lubec, Tadeusz Stefaniak, Ewa Willak-Janc, Józefa Chrzanowska

**Affiliations:** Department of Animal Products Technology and Quality Management, Wrocław University of Environmental and Life Sciences, ul. Chełmońskiego 37/41, 51-630 Wrocław, Poland; Department of Immunology, Pathophysiology and Veterinary Preventive Medicine, Wrocław University of Environmental and Life Sciences, Wrocław, Poland; Department of Pediatrics, Medical University of Vienna, Währinger Gürtel 18, 1090 Vienna, Austria; 1st Department and Clinic of Pediatric Allergology and Cardiology, Wrocław Medical University, M. Hoene-Wronskiego 13c, 50-376 Wroclaw, Poland

**Keywords:** Cow’s milk allergy, Whey protein hydrolysates, αs-casein hydrolysates, Cucurbita ficifolia, Serine protease

## Abstract

In the present study the effect of hydrolysis with non-commercial Cucurbita ficifolia serine protease on a reduction of the IgE and IgG binding capacity of whey protein concentrate and αs-casein was investigated. The intensity of the protein degradation was analyzed by the degree of hydrolysis, the free amino groups content and RP-HPLC. The ability to bind the antibodies by native proteins and their hydrolysates was determined using a competitive ELISA test. Deep hydrolysis contributed to a significant reduction of immunoreactive epitopes present in WPC. In the case of IgE and IgG present in the serum pool of children with CMA, the lowest binding capacity was detected in the 24 h WPC hydrolysate, where the inhibition of the reaction with native WPC was ≤23 and ≤60 %, respectively. The analysis of the IgG reactivity in the antiserum of the immunized goat showed that the lowest antibody binding capacity was exhibited also by 24 h WPC hydrolysate at a concentration of 1000 μg/ml where the inhibition of the reaction with nWPC was ≤47 %. One-hour hydrolysis of α-casein was sufficient to significant reduction of the protein antigenicity, while the longer time (5 h) of hydrolysis probably lead to the appearance of new epitopes reactive with polyclonal.

## Introduction

Food allergies, i.e. adverse reactions to food are an abnormal immune response to a specific food component. The reaction are known to cause many health problems (Koletzko et al. 2012) starting from skin reactions and hives, even to an anaphylactic shock. One of the most common is allergy that to milk and dairy products (cow’s milk allergy–CMA), which affects mainly children (Wal 2004; Kneepkens and Meijer 2009). It is reported that 0.6–2.5 % of preschoolers, 0.3 % of older children and teens, and up to 0.5 % of adults suffer from CMA (Fiocchi et al. 2010). When breast-feeding is not available or possible, cow’s milk is usually used as a natural substitute for human milk. Because milk proteins are the first exogenous proteins, such a substitution can lead to nutritional and immunological problems (Wal 2004; Kneepkens and Meijer 2009). CMA is mediated both through IgE and non-IgE mechanisms, where the non-IgE reactions are less readily recognized due to a less distinct temporal relationship between exposure and symptoms (Turnbull et al. 2015).

Cow’s milk contains two major protein fractions: casein and whey proteins. Casein is composed of α-S1-, α-S2-, β- and κ-fractions. While among whey proteins β-lactoglobulin (BLG, 55–60 %) and α-lactalbumin (15–20 %) are in the majority. Other minor proteins are bovine serum albumin, immunoglobulins and lactoferrin. Due to their outstanding nutritional traits and properties whey proteins are used as food ingredients in functional food products, such as infant formula, yogurt, meat and bakery products.

Studies conducted on large population of infants with CMA have shown, that the major milk allergens are BLG and α-S1-casein (Wal 2004; Mine and Yang 2008; Schulmeister et al. 2009). BLG—which is absent in human milk—may induce allergies in infants because of their underdeveloped gastrointestinal tract and immune system (Kattan et al. 2011). The BLG monomer is a globular protein composed of 162 amino acids with a molecular weight of 18.3 kDa. The tertiary structure of this globular protein, is stabilized by two disulfide bonds, makes it stable at low pH and resistant to gastric digestion (Lovegrove et al. 1993). In patients with persistent allergic reaction, seven IgE and six IgG binding epitopes were detected on BLG (Järvinen et al. 2001). Casein is, in general, easily digested, although some experiments (Schulmeister et al. 2009) have shown that some intact IgE-reactive fragments α-S1-casein that forms the core of the micelle are responsible for the induction of allergic reactions. It was also shown that the α-S1-casein found in human milk differs from the bovine casein (Otani et al. 1987).

Reduction or elimination of the major milk allergens by application of effective methods and technologies is essential to patients allergic to milk. However, it is very difficult to entirely remove the protein components from diet, and it is even harder to perform in case of infants who cannot be breast-fed (Kim et al. 2007).

Different attempts have been made to reduce the allergenicity of cow’s milk proteins and various technological processes have been applied for this purpose. Extensively hydrolyzed cow’s milk based formulas have been recommended; however, they have major drawbacks, such as an unacceptable bitter taste and high production costs (Bu et al. 2013). The remaining allergenicity depends on the degree of hydrolysis, the enzyme used and the technological processes such as filtration and heating (Bu et al. 2013). Thus, a study of the IgE responses to native, denatured and hydrolyzed cow’s milk proteins is critical for the development of new milk derivatives or replacements for sensitive patients.

Whey proteins are significantly resistant to being hydrolyzed. Proteases cleave milk proteins into peptides and may therefore have crucial effects on further gastrointestinal milk digestibility, release of the bioactive peptides, and exposure of antigenic epitopes (Raikos and Dassios 2014). The application of enzymes can increase the cost of the process, therefore cheap sources are preferred. Introducing enzymes derived from easily accessible sources may result in obtaining hydrolysates exhibiting potentially attractive properties, and simultaneously reducing production costs. For example, plant serine protease isolated from *C. ficifolia* fruit exhibits attractive proteolytic properties which have been analyzed towards e.g. casein or protein from corn gluten meal (CGM) (Illanes et al. 1985; Curotto et al. 1989).

The aim of this study was to investigate the effect of hydrolysis with non-commercial *C. ficifolia* serine protease on a reduction of the IgE and IgG binding capacity of whey protein concentrate and αs-casein.

## Materials and methods

*The enzyme* Serine protease was isolated from *C. ficifolia* fruit by the method of Dryjański et al. (1990). After separating peel and seeds, the pulp was homogenized and centrifuged 5000×*g*, 20 min. To the clear supernatant solid ammonium sulfate was added to the 30 % saturation. The final precipitate was collected by centrifugation at 5000 × *g*, 20 min. Desalting was conducted by dialysis in water. The specific activity of the enzyme preparation was 4411 U/g.

*Substrates* Whey protein concentrate (WPC-80) manufactured from sweet whey and spray dried was provided by Davisco Foods International, Inc. Alpha-s-casein was obtained according to the method of Thompson and Kiddy (1964) by a urea–calcium chloride procedure with a final precipitation in ethanol–ammonium acetate solution.

*Sera* All sera from patients were kindly provided by med. dr Ewa Willak-Janc from the 1st Department of Pediatrics, Allergy and Cardiology, Medical University in Wrocław.

*Hydrolysis of WPC-80 and αs- casein* Enzymatic hydrolysis of 1 % protein solution was conducted using serine protease isolated from *C. ficifolia* at the dose of 150 U/mg of hydrolyzed protein. The reaction was carried at 37 °C for 1, 3, 5, 24 h in 0.1 M Tris–HCl buffer at pH 8.0. The hydrolysis was terminated by thermal inactivation (for biological activity determinations) or by the addition of 10 % trichloroacetic acid (TCA) (1:1 V/V).

*The degree of hydrolysis* The course of the hydrolysis was monitored by the determination of soluble peptide concentration in 5 % TCA in relation to total protein. The concentration of the trichloroacetic acid-soluble product in the supernatant was measured spectrophotometrically at *λ* 280 nm (Spellman et al. 2003).

*The free amino groups’ concentration* The concentration of free amino groups (μmol Gly/g) was determined using trinitrobenzene sulfonic acid (TNBS, Sigma) according to the method described by Kuchro et al. (1983).

*Reversed-phase high-performance liquid chromatography (RP-HPLC)* Peptide profiles were determined by RP-HPLC with an Agilent 1100 Series system. The peptide preparations were solubilized in the even volume of phase A (0.1 % TFA in H_2_O) before loading on the chromatographic HPLC column (Zorbax Eclipse XDB-C18 Agilent column (50 × 4.6 mm). Separation was performed at a flow rate of 1 ml/min at 30 °C. Peptide fractions, varying in hydrophobicity, equal from the column in linear gradient of phase B (0.1 % TFA in acetonitrile). Absorbance measurement was made at *λ* = 230 nm (DAD, G1315B).

*Determination of protein content* Protein content was determined by colorimetric method of Lowry et al. (1951), using BSA (Sigma, P0834) as a standard.

### Determination of the binding capacity of IgG and IgE antibodies by WPC-80 hydrolysates

Analysis of antigenicity of WPC-80 hydrolysates. The ability to bind IgE and IgG antibodies by native WPC-80 (nWPC) and WPC-80 hydrolysates was determined using a competitive ELISA test according to the modified method described by Pescuma et al. (2011).

A pool of sera from 20 children (3–5 years old) with persistent cow’s milk allergy, as defined by a positive result in specific serum IgE was used in the study. Sera were selected from 37 analyzed samples and were characterized by the highest absorbance levels (OD_450nm_ ≥0.5) in the reaction with WPC. A pool of 5 sera, with high IgG reactivity (OD_450nm_ ≥0.5) with nWPC, taken from children not exhibiting any clinical symptoms of CMA was used as a control.

Maxi-Sorp 96-well plates (Nunc) were coated with 100 of 1 μg/ml nWPC solution in 0,1 M carbonate buffer (pH 9.5) per well. The plates were incubated for 2 h at 37 °C and then overnight at 4 °C. In the next step the plates were washed with PBST buffer (PBS pH = 7.4 with 0.05 % Tween 20) and blocked with a 2 % solution of Tween (300 μl per well) for 2 h at 37 °C. Before use, sera were preincubated (initial dilution 1:10 for determination of IgE reactivity, 1:3000 for IgG—present in the serum pool of the CMA children; 1: 2000 for IgG—in the serum pool of healthy children) with solutions of nWPC proteins or hydrolysates after 1, 3, 5, 24 of hydrolysis (starting protein concentrations were: 2000; 1000; 500; 100; 50; 10; 5; 2.5; 1.25; 0.63; 0.31; 0 μg/ml).

A pool of sera with various protein solutions was mixed at a ratio of 1:1 and incubated for 2 h at room temperature on rotary platform in 48-well plates. After incubation the mixture was loaded in triplicates on plates previously coated with nWPC solution and blocked and incubated for approximately 18 h at 4 °C on a rotary platform. As the secondary antibodies goat anti-human IgE conjugated to HRPO (Sigma-Aldrich A9667, dilution 1: 3000), or rabbit anti-human IgG conjugated to HRPO (Sigma-Aldrich A8792, dilution 1:60 000) were used in amount of 100 μl of conjugate per well. Afterwards the mixture was incubated for 1.5 h at room temperature with gentle rotation. The reaction was developed for 30 min using the TMB Super Sensitive substrate (Sigma-Aldrich, T4444). Readings were performed at 450 nm using a Quantum reader (Biotek).

The percentage of antigen-binding inhibition was calculated using the following equation:

Inhibition rate (%) = [(Abs_o_−Abs_x_)*/*Abs_o_] × 100, where Abs_o_ was the mean absorbance value of no-antigen triplicates (serum was incubated with PBST at a ratio of 1:1); Absx was the mean absorbance value of triplicates obtained with different antigen concentrations.

### Determination of the binding capacity of goat anti-whey IgG/immune sera by WPC hydrolysates

The binding capacity of goat anti-whey IgG by WPC-80 hydrolysates was determined using the competitive ELISA test. The analyzed immune serum obtained as a result of 5-time, intradermal immunization of goats with whey proteins. The obtained and purified antigenic preparations, emulsified with Freund’s complete adjuvant (Calbiochem) were administered every 14 days. Twelve days after the last injection, blood was collected from the cervical vein and the obtained serum was stored at −20 °C until use (Stefaniak et al. 2000).

The experiment was conducted as described in previous section. The goat immune serum, diluted 1:15 000, was preincubated with nWPC/WPC hydrolysates obtained after 1, 3, 5, 24 h of hydrolysis. Secondary antibodies used in the test were rabbit anti-goat IgG conjugated to HRPO (Sigma-Aldrich A5420, dilution 1:20,000).

### Determination of the binding capacity of IgG and IgE antibodies by αs-casein hydrolysates

The binding capacity of IgE and IgG by native αs-casein (nα-c) and αs-casein hydrolysates was determined using a competitive ELISA.

A pool of sera from 20 children (3–5 years old) with persistent cow’s milk allergy, as defined by a positive result in a specific serum IgE was used in the study. Sera were selected from 37 analyzed samples and were characterized by the highest absorbance levels (OD_450nm_ ≥ 0.5) in the reaction with nα-c. A pool of 5 sera, with high IgG reactivity (OD_450nm_ ≥ 0.5) with nα-c was taken from children not exhibiting any clinical symptoms of CMA was used as a control.

The experiment was conducted as described above. In the pre-incubation of sera with nα-c protein solutions/α-c hydrolysates after 1, 5, 24 h of hydrolysis, the following starting dilutions of serum pool were used: 1:10 in the case of determining the IgE reactivity and 1:3000 for IgG in the group of children with CMA, and 1:2000 in the group of healthy children.

### Statistical analysis

All assays were conducted in triplicate. The results were analyzed using an Statistica 7.0 program analysis of variance (ANOVA), followed by a Duncan multiple range test to determine the significant difference between sample at *p* ≤ 0.05.

## Results and discussion

The elimination of all cow’s milk products, without appropriate substitutions, can lead to malnutrition and/or specific nutrient deficiencies at a time when infants and children are growing. Hence there is a need for reduction or elimination these major milk allergens by effective methods and technologies. Proteolysis may be considered an efficient way of removing allergenic epitopes in proteins and increasing their digestibility (Jędrychowski and Wróblewska 1999; Wróblewska et al. 2004). Based on the results of a randomized controlled study, only an extensively hydrolyzed formula was able to significantly decrease the prevalence of CMA (Businco et al. 1999). Extensively hydrolyzed protein formula, rather than an amino acid formula, is recommended for infants with IgE mediated CMA at low risk of anaphylactic reactions (no prior history of anaphylaxis or currently receiving an extensively hydrolyzed protein formula) (Koletzko et al. 2012).

### Extent of hydrolysis

**Fig. 1:**
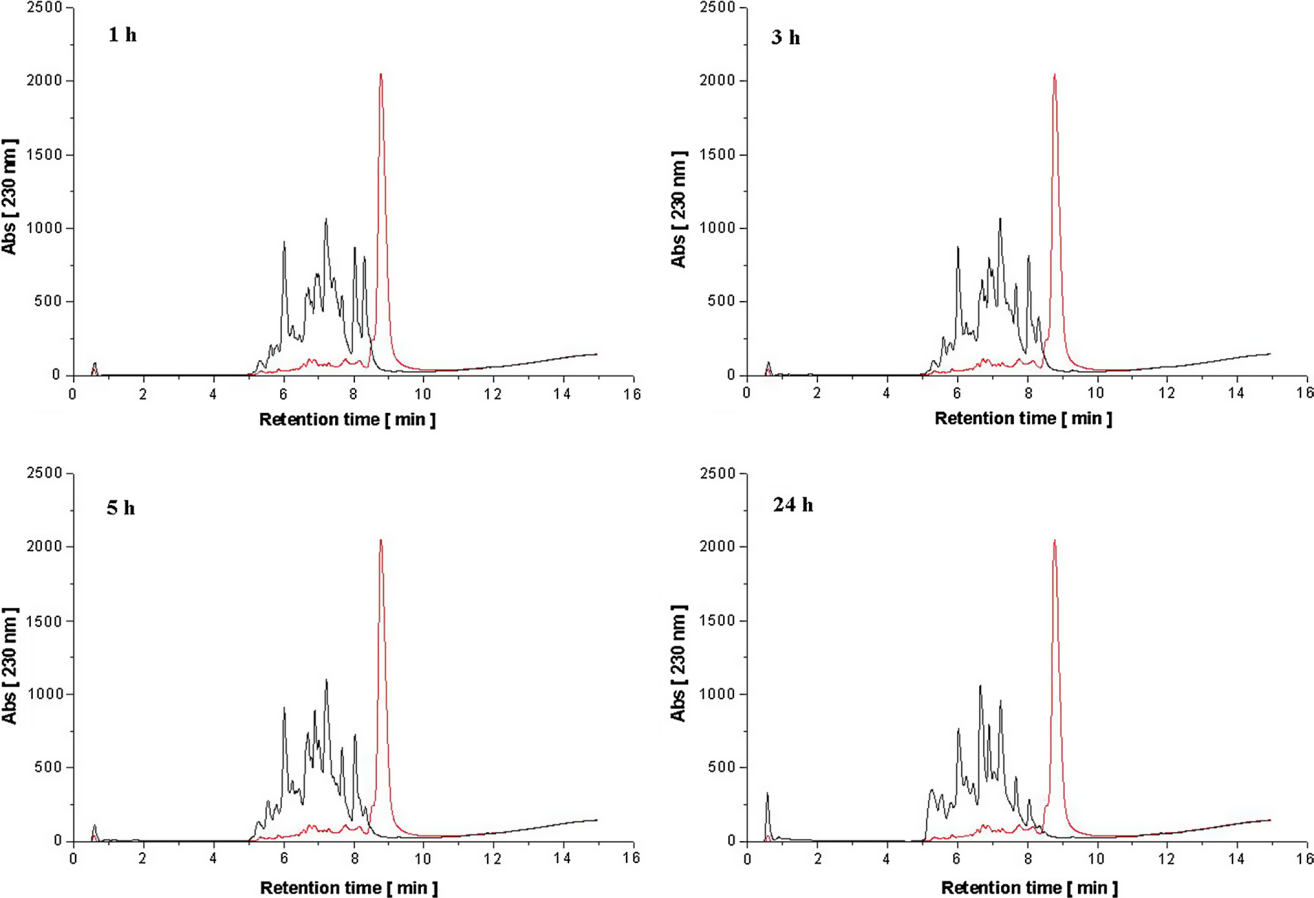
RP-HPLC profiles of peptide fractions (*black*) obtained after 1, 3, 5, 24 h hydrolysis of αs-casein with serine protease isolated from *Cucurbita ficifolia* introduced at a dose of 150 U/mg. Undigested 1 % protein solution was used as control (*red*) (color figure online)

**Fig. 2:**
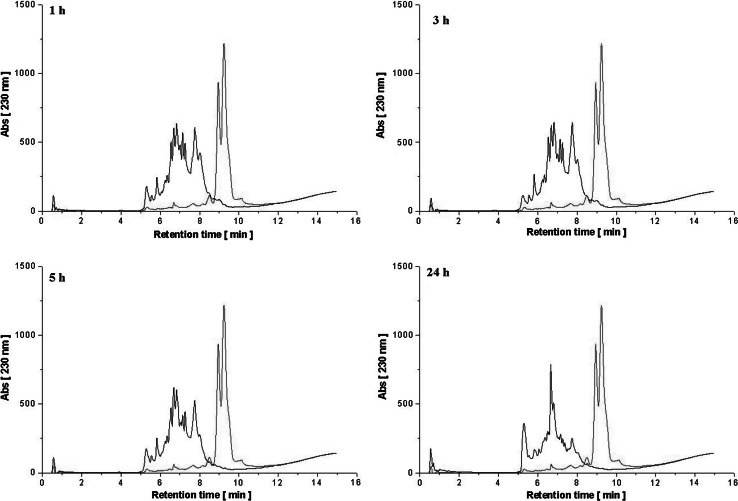
RP-HPLC profiles of peptide fractions (*black*) obtained after 1, 3, 5, 24 h hydrolysis of WPC-80 with serine protease isolated from *Cucurbita ficifolia* introduced at a dose of 150 U/mg. Undigested 1 % protein solution was used as control (*red*) (color figure online)

The course of the enzymatic hydrolysis of WPC-80 and αs-casein was monitored by determination of the degree of hydrolysis (DH) (%) (Table 1). The determined DH for αs-casein and WPC-80 after 24 h hydrolysis reached the values of 61.3 and 43.2 %, respectively. During the protein degradation in all hydrolysates, a proportional increase in free amino group concentration (FAG) was also observed (Table 1). The final concentrations of determined FAG reached 4725 µmol Gly/g for αs-casein hydrolysate and 4142 µmol Gly/g for WPC-80 hydrolysate. The progress of hydrolysis was also confirmed by RP-HPLC peptide profiles analysis (Figs. 1, 2). The presence of peptide fractions, eluted from the column at low concentration of acetonitrile and differing in terms of hydrophobicity, was noted on all hydrolysates chromatograms. BLG, the most abundant protein present in bovine whey, is known for its low susceptibility to enzymatic degradation. The RP-HPLC separation showed that even 1 h of hydrolysis with *C. ficifolia* serine protease resulted in almost complete disappearance of the peaks originating from the substrate. The use of this enzyme offers the possibility for protein degradation without other additional technological treatments, such as thermal and/or high pressure processing. Similar effects were observed by Ena et al. (1995), who studied conformation changes of proteins in WPC preparations after their hydrolytic degradation. During 3 h hydrolysis with Corolase 7092 (peptidases from *Aspergillus* strains) particular fractions were almost undetectable with the SDS-PAGE method. 24 h hydrolysis resulted in DH exceeding 21 %. Also Vázquez-Lara et al. (2003) demonstrated that 2 h hydrolysis of native β-lactoglobulin with the plant protease Actinidin lead to a DH of 43 %.

**Table 1:** Degree of hydrolysis DH (%) and free amino groups concentration FAG (µM Gly/g) in hydrolysates obtained using serine protease isolated from *Cucurbita ficifolia*

Time of hydrolysis (h)	WPC-80				αs-Casein			
	FAG (µmol Gly/g)	SEM	DH (%)	SEM	FAG (µmol Gly/g)	SEM	DH (%)	SEM
1	1816 a	16.39	18.96 a	0.74	2627 a	44.57	34.07 a	1.36
3	2237 b	8.2	23.35 b	0.91	2837 b	39.99	36.79 b	0.34
5	2924 c	26.06	30.53 c	1.15	3885 c	35.15	50.39 c	0.51
24	4142 d	43.96	43.23 d	0.96	4725 d	20.88	61.27 d	0.73

All data were expressed as mean values (*n* = 3)

Mean values with different letters in the same column are significantly different at *p* < 0.05

*SEM* the standard error of the mean

### Antigenicity of WPC hydrolysates

**Fig. 3:**
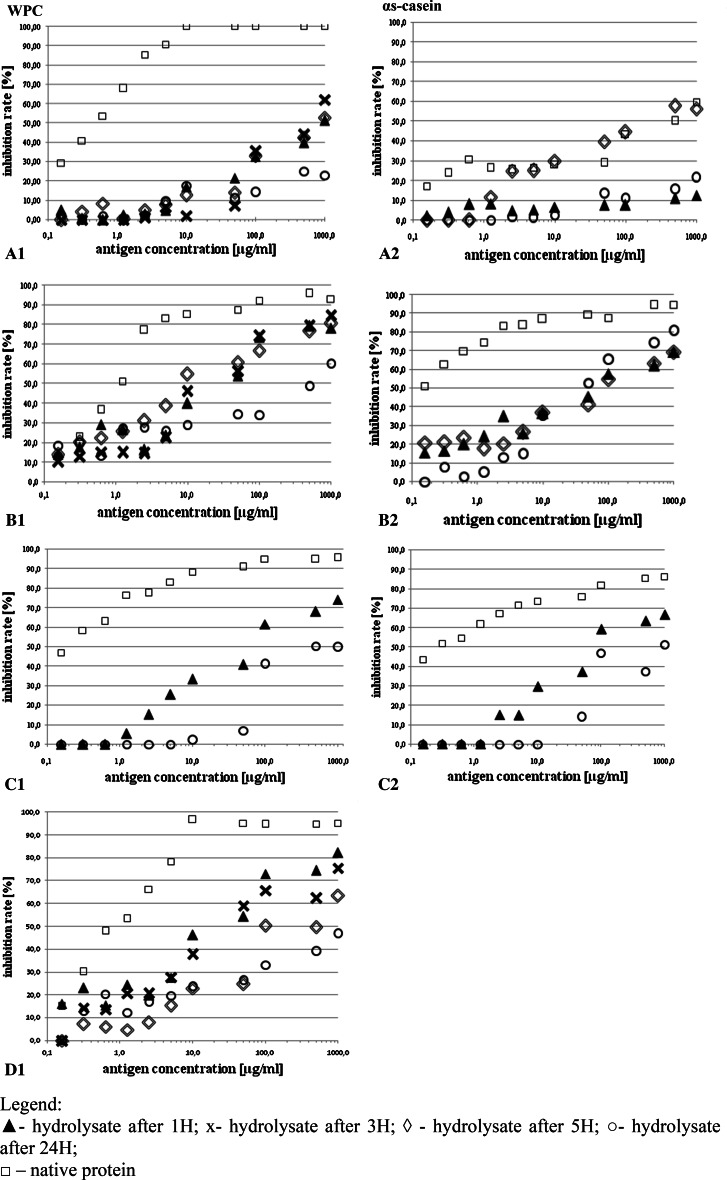
Effect of pre-incubation of the sera pool from children with CMA (**a**, **b**), healthy children (**c**), goat antiserum (**d**), with a native protein/hydrolysed WPC (**.1**) and αs-casein (**.2**) on the inhibition of the reactivity of IgE (**a.1**, **a.2**) or IgG (**b.1**, **b.2**, **c.1**, **c.2**, **d1**)

Analysis of the IgE reactivity in the sera of children with cow’s milk allergy (Fig. 3a.1) showed that a 2-hour pre-incubation of antibodies with nWPC (at a concentration ≥10 μg/ml) induced the binding of all WPC-specific IgE antibodies. Hydrolysis resulted in a reduction (approx. 24–77 %) in IgE-binding capacity by peptides as compared to the control (nWPC). It was also observed that prolonged hydrolysis did not result in a decrease in binding capacity of IgE by ≤50 μg/ml solution of WPC hydrolysates. For all hydrolysates with those concentrations we observed less than 20 % inhibition of IgE antibody reactivity with nWPC.

This observation may suggest that the obtained hydrolysates have the ability to bind similar amounts of antibodies, and thus the degree of degradation did not reduce the amount of reactive epitopes. It should be noted however, that this observation may be due to the competition for binding sites (epitopes) between antibodies of different classes, i.e.: IgE, IgG and IgM present in the analyzed samples.

The competition for epitopes may interfere with the reactivity of IgE antibodies, particularly as the concentration of these antibodies in the serum is much lower than the concentration of IgG. At higher hydrolysates concentrations (>50 μg/ml), where the number of potential sites for antibody binding was higher, the lowest IgE-binding capacity was detected in the 24 h WPC hydrolysate, where the inhibition of the reaction with nWPC was ≤23 %. Deep hydrolysis contributed to a reduction of higher amounts of reactive epitopes present in WPC.

The research by Pescuma et al. (2011) indicates that the hydrolysis of BLG by *Lactobacillus* strain, at the concentration of 1 mg/ml, considerably reduced (32 %) its recognition by the IgE of allergic children as compared to heat-denatured BLG. Although the degradation of this protein was incomplete, the IgE-binding reaction was still observed.

In the case of IgG present in the serum pool of children with CMA (Fig. 3b.1) a high binding capacity of this antibody by both the control and the obtained hydrolysates was determined. In the case of the highest concentrations of analyzed WPC hydrolysates (1000 µg/ml), we found no significant differences in the IgG-binding capacity between the control and the hydrolysates obtained after 1, 3 and 5 h of hydrolysis. Inhibition of reactivity with nWPC did not exceed 78 %. The lowest IgG-binding capacity was determined in 24 h nWPC hydrolysate characterized by the highest degree of degradation, where the inhibition of the reaction with nWPC was ≤60 %.

Analysis of the reactivity of IgG present in the sera of healthy children (Fig. 3c.1) showed, as in the case of children with allergy, a high binding capacity for IgG by nWPC. There was a significant reduction (in the range of 21.5–84 %) in binding capacity for IgG by 1 h and 24 h hydrolysates compared to the control. Hydrolysate obtained after 24H of proteolysis, at a concentration of ≤10 μg/ml, showed no significant reactivity for IgG in the ELISA. It can be assumed that the application of this type of hydrolysate to an infant would not raise their immune system response.

It has been shown that exposure to cow milk protein during the first 3 months of life results in high levels of IgG subclass antibodies, detected even at 8 years of age (Jenmalm and Björkstén 1998). Similar findings were reported by Nentwich et al. 2004 who demonstrated the presence of specific IgG antibodies against cow’s milk protein not only in the serum of children and an adult with CMA, but also in the serum of healthy people consuming cow’s milk. It was demonstrated that CMA patients are characterized by elevated levels of specific IgE levels as well as the specific IgG levels (Shek et al. 2005; Meulenbroek et al. 2013). The IgG antibody could play an important role in allergen-Ab complex formation. The presence of IgG in allergen–IgE complexes may result in binding to B cells which may affect Ag processing and presentation, and thereby influencing the allergic response (Meulenbroek et al. 2013).

Oldaeus et al. 1999 analyzed the presence of IgG and IgE antibodies specific to BLG in serum samples of 94 infants with a family allergy medical history, who had been fed with cow’s milk formulas of different DH. They showed that the IgG responses to BLG were very low in the group exposed to extensively hydrolyzed formula, intermediate in the partially hydrolyzed formula, and high in case of a regular cow’s milk formula. High immune responses connected to high concentration of specific IgE and IgG antibodies were associated with the development of atopic disease. The low antigenicity and allergenicity of the extensively hydrolyzed formula supports its use in allergy prophylaxis.

The analysis of the IgG antibodies reactivity in the antiserum of the immunized goat (Fig. 3d.1) showed that a 2-h pre-incubation with nWPC antibodies (at a protein concentration = 1000 μg/ml) contributed to nearly 100 % of inhibition of their reactivity, while a 24-h pre-incubation with the hydrolysate at the same concentration caused inhibition of the reaction at the two times lower level.

In the case of using low concentrations of hydrolysates (<5 μg/ml) longer hydrolysis did not decrease the binding capacity for anti-WPC antibodies by the resulting peptides. For all hydrolysates it was found that the inhibition of antibody reactivity for nWPC at a similar level (≤30 %). A difference was found for higher concentrations of hydrolysates (≥5 μg/ml), where the progress of hydrolysis reduced the antigenicity of the whey proteins. The lowest antibody binding capacity was exhibited by hydrolysates 5 h and 24 h, at a concentration of 1000 μg/ml the inhibition of the reaction with nWPC was ≤63.4 and ≤47 %, respectively. This experiment confirmed that the hydrolysis procedure caused a partial reduction in reactive epitopes, which lead to a reduction of antigenicity of the WPC proteins.

### Antigenicity of αs-casein hydrolysates

Analysis of the reactivity of IgE antibodies present in the serum pool of children with an allergy to cow’s milk (Fig. 3a.2) showed that 2-hour pre-incubation of antibodies with nα-c did not lead to a binding of all IgE specific for that protein. The inhibition of the reaction was ≤60 %. The observed phenomenon could have been caused by the strong competition for free epitopes between different classes of antibodies; some epitopes were bound by both IgG and IgM.

The time of hydrolysis caused a decrease in IgE-binding capacity of the resulting peptides compared to control, but the most preferred effect was observed after the 1 and 24 h hydrolyses, but not after the 5-hour hydrolysis (inhibition of reaction with nα-c was respectively ≤12, ≤21 ≤ 56.4 %). One-hour hydrolysis of α-casein was sufficient to significant reduction of the protein antigenicity, while the longer time (5 h) of hydrolysis probably lead to the appearance of new epitopes reactive with polyclonal anti- αs-casein IgE which were then degraded by further hydrolysis (24 h).

To date, the presence of nα-c specific IgG and IgE has been demonstrated in the serum of patients with CMA, as well as IgG antibodies in healthy individuals (Nentwich et al. 2004; Meulenbroek et al. 2013). It has been proven, however, that among the three fractions of casein, alpha-casein has the lowest reactivity with IgG antibodies present in the serum of patients with CMA (Nentwich et al. 2004).

As shown by other researchers, hydrolysis can either decrease or increase cow’s milk protein immunoreactivity as it is directly dependent on the specific activities of the different enzymes (Wróblewska and Kaliszewska 2012). During hydrolysis the protein is cleaved to different in size peptide fragments. Their properties are dependent from the amino acid sequence of the protein and also from their secondary structure. Hydrolysis of BLG by trypsin/chymotrypsin reduces its allergenicity, but also exposes hidden allergenic peptides, which are recognized by the specific IgE of allergic patients (Selo et al. 1999).

Analysis of the reactivity of IgG present in the serum pool of children with an allergy to cow’s milk (Fig. 3b.2) showed a high binding capacity for this class of antibodies by nα-c. The resulting hydrolysates showed lower in comparison to control inhibition of reactivity with nα-c. However, the prolonged hydrolysis did not result in the decrease of binding capacity for IgG antibodies by the peptides (maximum difference in the inhibition of reactivity with nα-c between the 1H and 24H hydrolysates did not exceed 20 %). At a hydrolysates concentration of 1000 μg/ml the lowest binding capacity of IgG antibodies was found in hydrolysates after 1 and 5 h of the process, where reactivity inhibition in those samples was about 25 % lower than the controls. This can be partly explained by the altered aggregation and immunologic behavior of peptides in the entire hydrolysate mixture, where especially in high concentrations, they tend to re-associate and form macromolecular complexes.

In our study, analysis of the reactivity of IgG present in the serum pool of healthy children (Fig. 3c.2) showed a high binding capacity for this class of antibodies by nα-c. The lowest binding capacity of IgG antibodies specific for nα-c was shown by hydrolysate 24H, and the observed inhibition of the reaction with nα-c compared to control was at least 35 % lower. In the research by Nentwich et al. (2004), casein hydrolysate demonstrated only about 18.5 % casein antigenicity. No significant difference in IgG binding was detected between the patient and control sera in casein hydrolysate.

## Conclusion

The results obtained in the present study demonstrate that hydrolysis using a non-commercial serine protease isolated from *C. ficifolia* led to a significant decrease in the antigenic IgE and IgG response to major allergens from bovine milk (WPC and αs-casein). These findings suggest that hydrolysates with such traits may have the potential to be administrated in the prophylactic treatment of infants with a high risk of allergy.
